# T‐LAK cell‐originated protein kinase (TOPK): an emerging prognostic biomarker and therapeutic target in osteosarcoma

**DOI:** 10.1002/1878-0261.13039

**Published:** 2021-06-29

**Authors:** Pichaya Thanindratarn, Ran Wei, Dylan C. Dean, Arun Singh, Noah Federman, Scott D. Nelson, Francis J. Hornicek, Zhenfeng Duan

**Affiliations:** ^1^ Department of Orthopaedic Surgery Sarcoma Biology Laboratory David Geffen School of Medicine University of California Los Angeles CA USA; ^2^ Department of Orthopedic Surgery Chulabhorn Hospital HRH Princess Chulabhorn College of Medical Science Chulabhorn Royal Academy Bangkok Thailand; ^3^ Musculoskeletal Tumor Center Beijing Key Laboratory of Musculoskeletal Tumor Peking University People’s Hospital Beijing China; ^4^ Sarcoma Service Division of Hematology‐Oncology David Geffen School of Medicine at UCLA Los Angeles CA USA; ^5^ Department of Pediatrics Mattel Children’s Hospital David Geffen School of Medicine at UCLA Los Angeles CA USA; ^6^ UCLA’s Jonsson Comprehensive Cancer Center Los Angeles CA USA; ^7^ Department of Pathology University of California Los Angeles CA USA

**Keywords:** immunohistochemistry, osteosarcoma, prognostic biomarker, TOPK

## Abstract

T‐lymphokine‐activated killer (T‐LAK) cell‐originated protein kinase (TOPK) is an emerging target with critical roles in various cancers; however, its expression and function in osteosarcoma remain unexplored. We evaluated TOPK expression using RNA sequencing and gene expression data from public databases (TARGET‐OS, CCLE, GTEx, and GENT2) and immunohistochemistry in an osteosarcoma tissue microarray (TMA). TOPK gene expression was significantly higher in osteosarcoma than normal tissues and directly correlated with shorter overall survival. TOPK was overexpressed in 83.3% of the osteosarcoma specimens within our TMA and all osteosarcoma cell lines, whereas normal osteoblast cells had no aberrant expression. High expression of TOPK associated with metastasis, disease status, and shorter overall survival. Silencing of TOPK with small interfering RNA (siRNA) decreased cell viability, and inhibition with the selective inhibitor OTS514 suppressed osteosarcoma cell proliferation, migration, colony‐forming ability, and spheroid growth. Enhanced chemotherapeutic sensitivity and a synergistic effect were also observed with the combination of OTS514 and either doxorubicin or cisplatin in osteosarcoma cell lines. Taken together, our study demonstrated that TOPK is a potential prognostic biomarker and therapeutic target for osteosarcoma treatment.

## Graphical abstract

TOPK is aberrantly expressed in osteosarcoma and significantly correlates with shorter overall survival (OS). Therapeutic inhibition of TOPK decreases osteosarcoma cell growth, proliferation, migration, and colony formation. These findings indicate TOPK as a prognostic biomarker for OS and a potential therapeutic target in patients with osteosarcoma.

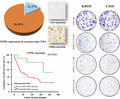

**Abbreviations**
3D: three‐dimensional
ANOVA: A one‐way analysis of variance
ATCC: the American Type Culture Collection
CCLE: the Cancer Cell Line Encyclopedia
CI: confident interval
DMSO: dimethyl sulfoxide
FBS: fetal bovine serum
FFPE: formalin‐fixed paraffin‐embedded
GENT2: the Gene Expression database of Normal and Tumor tissue 2 database
GTEx: the Genotype‐Tissue Expression project
HR: hazard ratio
IC50: half-maximal inhibitory concentration
IHC: immunohistochemistry
MAPKK: mitogen-activated protein kinase (MAPK) kinase
mRNA: messenger RNA
MTT: conventional 3-(4,5-dimethylthiazol-2-yl)-2,5-diphenyl tetrazolium bromide assays
OS: overall survival
PARP: poly (ADP-ribose) polymerase
PBK: PDZ-binding kinase
RFS: recurrence-free survival
shRNA: short hairpin RNA
siRNA: small interfering RNA
TARGET-OS: the Therapeutically Applicable Research to Generate Effective Treatment on Osteosarcoma
TMA: tissue microarray
TOPK: T-lymphokine-activated killer cell-originated protein kinase
TPM: Transcripts per million unit
ZIP: zero interaction potency model

## 1 Introduction

Osteosarcoma is the common primary malignancy of the bone, disproportionately affects children and adolescents, yet accounts for < 1% of all cancer diagnoses within the United States [1]. While most arise within the metaphysis of long bones, axial lesions do occur and tend to be more aggressive [2]. Despite an aggressive approach and often burdensome treatment, the 5‐year overall survival rate has plateaued at approximately 67% for nonmetastatic osteosarcoma, with no significant progress in the past four decades especially for those with recurrence, metastasis, or cytotoxic drug resistance [2, 3]. Despite increasing efforts of targeted therapy for osteosarcoma, including some tyrosine kinase inhibitors (pazopanib, sorafenib, and regorafenib), these agents have failed to improve patient outcomes [4, 5, 6]. Several cancer immunotherapeutics including immune checkpoint inhibitors have been investigated as well; however, their efficacy has been dampened by the prominent heterogeneity of receptors and tumor microenvironment within osteosarcoma [7, 8]. The limitations of these approaches highlight the urgent need for novel therapeutic strategies.

Recent works have shown T‐lymphokine‐activated killer (T‐LAK) cell‐originated protein kinase (TOPK) is instrumental in the pathogenesis of various cancers [9, 10, 11, 12]. Also called PBK (PDZ‐binding kinase), this is a 322‐amino‐acid serine/threonine kinase encoded by the PBK gene located on chromosome 8p21.1. When activated, it functions as a mitogen‐activated protein kinase (MAPK) kinase (MAPKK) essential for cell division [9]. TOPK has extensive mitotic roles and is a regulator of numerous DNA‐binding proteins [13]. It is expressed mainly in the cytoplasm [9]. While TOPK expression is low to undetectable in normal tissues [9], higher expression exists in various human cancers including lung cancer, colorectal cancer, ovarian cancer, kidney cancer, prostate cancer, and hematologic malignancies [11, 14, 15, 16, 17, 18, 19]. TOPK has garnered clinical interest as heightened expression correlates with poor clinical outcomes [11, 14, 15, 16, 17, 18, 19]. Functionally, TOPK sustains cancer cell growth and proliferation, and tumor dissemination, and enhances apoptotic resistance [9]. In addition, it is upregulated in cancer stem cells where it promotes their proliferation and self‐renewal in various malignancies [20, 21]. Accumulating research supports TOPK as an emerging prognostic biomarker and therapeutic target that is highly specific for cancer cells, which has prompted the development of several specific and potent inhibitors of TOPK. These inhibitors have shown encouraging results in preclinical cancer models and will likely move to the clinical trial phase within the near future.

Given the encouraging results of TOPK targeting in other cancers and the limitations of current osteosarcoma regimens, we investigated the following: (a) expression of TOPK in public databases, with additional validation of this expression in human osteosarcoma tissues and cell lines; (b) correlation between TOPK expression and clinicopathology and outcomes of osteosarcoma patients; (c) functions of TOPK in osteosarcoma cell growth and proliferation; and (d) effects of a specific TOPK inhibitor on osteosarcoma cell proliferation, migration, and chemosensitivity.

## 2 Materials and methods

### 2.1 TOPK gene expression and RNA sequencing data from public databases

**Fig. 1:**
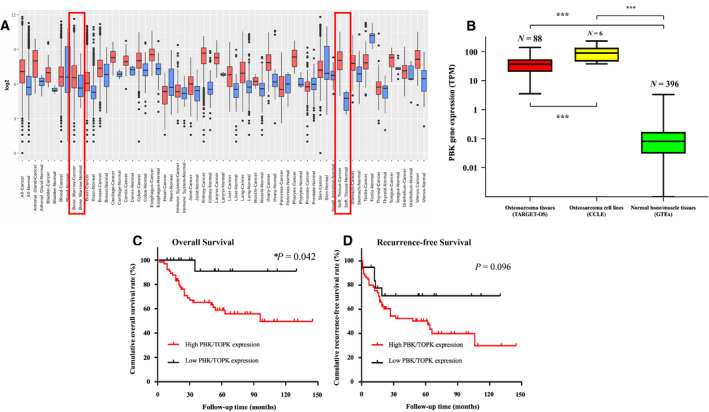
Evaluation of TOPK gene expression in normal and cancer tissues via public database. (A) Boxplot shows the tissue‐wide gene expression pattern of TOPK across paired tissue samples from the GPL96 platform (HG‐U133A) of Affymetrix mRNA gene array GENT2 database. Red indicates boxplot of cancer samples and blue indicates boxplot of normal samples. (B) Gene expression of TOPK in osteosarcoma tissues (TARGET‐OS), cell lines (CCLE), and normal bone or muscle tissue (GTEx). The data exhibits the mean ± SEM of the experiment. Mann–Whitney *U*‐test was used to analyze statistical significance. ***P < 0.001. (C) Kaplan–Meier overall survival curve of osteosarcoma patients in the TARGET‐OS database, subgrouped into either the low or high TOPK gene expression groups. Compared with the low TOPK gene expression group, patients with high TOPK gene expression had significantly shorter overall survival (*P* = 0.042) by Cox regression analysis. (D) Kaplan–Meier recurrence‐free survival curve of osteosarcoma patients in the TARGET‐OS database were subgrouped in a similar fashion as Fig. 1C. Compared with the low TOPK gene expression group, patients with high TOPK gene expression tended to have shorter recurrence‐free survival but was not statistically significant by Cox regression analysis.

Publicly available genomic databases with their immense DNA and RNA sequencing data have streamlined the identification of aberrantly expressed genes and their molecular mechanisms driving tumor progression. Expression of the TOPK gene across various tumor tissues was contrasted to their healthy controls within the Gene Expression database of Normal and Tumor tissue 2 (GENT2) database [22] at https://gent2.appex.kr/gent2/ (Fig. 1A). The Therapeutically Applicable Research to Generate Effective Treatment on Osteosarcoma (TARGET‐OS) is a comprehensive genomic database which serves to delineate the molecular changes driving osteosarcoma. The TARGET‐OS database contains comprehensive genomic profiles of clinically annotated patient cases within the discovery dataset. Each fully characterized TARGET‐OS case includes data from nucleic acid samples extracted from osteosarcoma tissue. In our study, RNA sequencing data of TOPK in osteosarcoma tissues were obtained from TARGET‐OS (https://portal.gdc.cancer.gov/projects/TARGET‐OS) and downloaded from the UCSC Xena browser at https://xenabrowser.net. As the control, normal bone and muscle tissue expressions of TOPK from RNA sequencing were obtained from the Genotype‐Tissue Expression (GTEx) project [23]. The expressions of TOPK in osteosarcoma cell lines were collected from the Cancer Cell Line Encyclopedia (CCLE) [24] (Fig. 1B). Transcripts per million unit (TPM) was used to compare TOPK gene expression from RNA sequencing [25].

### 2.2 Osteosarcoma sample collection and tissue microarray (TMA)

TMA was made from 66 individual osteosarcoma patient tissues within a formalin‐fixed paraffin‐embedded (FFPE) block as previously described [26]. Our study methodologies were conformed to the standard set by the Declaration of Helsinki and approved by the ethics committee. The clinical characteristics of the tissue specimens were collected and showed in Table 1, including gender, age, tumor location, histologic grade, disease status, and follow‐up time. The samples included 40 (60.6%) males and 26 (39.4%) females with an average age of 31.5 years (range: 6–77 years old). The mean follow‐up time was 94.1 months (range: 0–273 months). The most common tumor location was the femur (48.5%), followed by the tibia (16.7%), humerus (12.1%), pelvis and vertebrae (12.1%), and others (10.6%). Of the 66 patients, 22 (33.3%) developed recurrence and 47 (71.2%) developed metastasis.

**Table 1:** Correlations between TOPK expression and clinicopathology in osteosarcoma patients.

Clinicopathological features	Number of cases	TOPK expression		*P* value
	(*n*, %)	Low (*n*, %)	High (*n*, %)	
All patients	66 (100.0)	11 (16.7)	55 (83.3)	
Age (years)				
< 20	23 (34.8)	4 (17.4)	19 (82.6)	0.966
20–60	38 (57.6)	6 (15.8)	32 (84.2)	
> 60	5 (7.6)	1 (20.0)	4 (80.0)	
Gender				
Male	40 (60.6)	7 (17.5)	33 (82.5)	0.822
Female	26 (39.4)	4 (15.4)	22 (84.6)	
Tumor location				
Femur	32 (48.5)	5 (15.6)	27 (84.4)	0.442
Tibia	11 (16.7)	0 (0.0)	11 (100.0)	
Humerus	8 (12.1)	2 (25.0)	6 (75.0)	
Pelvis and vertebrae	8 (12.1)	2 (25.0)	6 (75.0)	
Others	7 (10.6)	2 (28.6)	5 (71.4)	
Histologic grade				
Low grade	9 (13.6)	2 (22.2)	7 (77.8)	0.630
High grade	57 (86.4)	9 (15.8)	48 (84.2)	
Recurrence				
Absent	44 (66.7)	9 (20.5)	35 (79.5)	0.243
Present	22 (33.3)	2 (9.1)	20 (90.9)	
Metastasis				
Absent	19 (28.8)	6 (31.6)	13 (68.4)	0.039*
Present	47 (71.2)	5 (10.6)	42 (89.4)	
Disease status				
No evidence of disease	23 (34.8)	7 (30.4)	16 (69.6)	0.046*
Alive with disease	3 (4.6)	1 (33.3)	2 (66.7)	
Died of disease	40 (60.6)	3 (7.5)	37 (92.5)	

The tumor tissues in this TMA were derived from both primary and recurrent tumors. The metastatic specimens mean for patients with metastatic diseases, including patients with primary metastatic disease and patients who developed metastatic relapse. The term ‘recurrence’ referred to any local recurrence during follow‐up, while ‘metastasis’ referred to development of distant metastasis during follow‐up.

* Statistically significant.

### 2.3 Immunohistochemistry (IHC)

The expression of TOPK was accessed in an IHC assay with TOPK antibody according to manufacturer instructions (Cell Signaling Technology, Danvers, MA, USA). In brief, the paraffin‐embedded slide was first baked at 60 °C for 1 h before deparaffinization with xylene. The slide was then re‐hydrated via graded ethanol (100% and 95%). Following heated epitope retrieval, 3% hydrogen peroxide (H_2_O_2_) was used to dampen endogenous peroxidase activity. The slide was then blocked with normal goat serum for 1 h. Subsequently, the slide was incubated with antibody to human TOPK (1 : 100 dilution, Cell Signaling Technology) in a humidified chamber at 4 °C overnight. Then, SignalStain^®^ Boost Detection Reagent (Cell Signaling Technology) and SignalStain^®^ DAB (Cell Signaling Technology) were applied to detect the bound antibody. Hematoxylin QS (Vector Laboratories, Burlingame, CA, USA) was used to counterstained all of the sections, and the slide was finally mounted with VectaMount AQ (Vector Laboratories) for long‐term preservation.

**Fig. 2:**
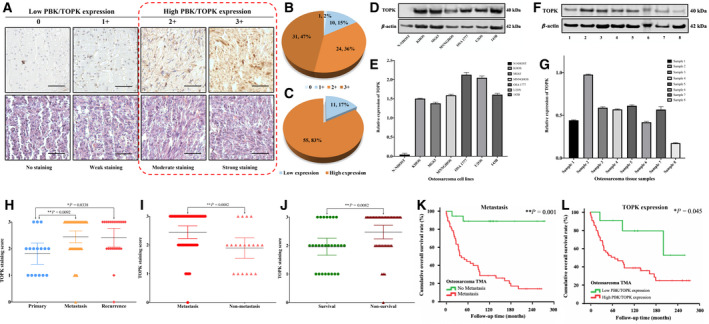
Evaluation of TOPK expression and staining in an osteosarcoma TMA, fresh tissues, and cell lines. (A) Representative images of different immunohistochemistry staining intensities of TOPK and H&E staining are shown in osteosarcoma tissues. Based on the TOPK staining in the tumor samples, the cytoplasmic staining patterns were divided into 4 groups: no staining (0); weak staining (1+); moderate staining (2+); strong staining (3+). (Original magnification, 400×; Scale bar, 50 μm). The data exhibit independent triple specimens from each patient in this TMA. (B) Pie chart representing relative frequency of different TOPK expression levels in osteosarcoma tissue microarrays. (C) Pie chart representing relative frequency of high TOPK expression (staining score ≥ 2+) and low TOPK expression (staining score ≤ 1+) in osteosarcoma tissue microarrays. (D) Differential expression of TOPK in various osteosarcoma cell lines, along with a normal osteoblast cell line via western blot. (E) Densitometry quantification of western blot results from Fig. 2D, presented as relative to β‐actin expression. The data exhibit the mean ± SD of the experiment carried out in triplicate. (F) Expression of TOPK in osteosarcoma tissue samples from eight patients via western blot. (G) Densitometry quantification of western blot results from Fig. 2F, presented as relative to β‐actin expression. The data exhibit the mean ± SD of the experiment carried out in triplicate. (H) Distribution of TOPK immunostaining scores in primary osteosarcoma tissues compared with tissues from patients with recurrent and metastatic disease. The error bars indicate SEM with 95% CI of each group. Mann–Whitney *U*‐test was used to analyze statistical significance. ***P < 0.001. (I) Comparison of TOPK immunohistochemistry staining scores between osteosarcoma tissues from patients with metastatic and nonmetastatic disease at the end of follow‐up time. The error bars indicate SEM with 95% CI of each group. Mann–Whitney *U*‐test was used to analyze statistical significance. ***P < 0.001. (J) Comparison of TOPK immunohistochemistry staining scores between osteosarcoma tissues from survival and nonsurvival patients at the end of follow‐up time. The error bars indicate SEM with 95% CI of each group. Mann–Whitney *U*‐test was used to analyze statistical significance. ***P < 0.001. (K) Kaplan–Meier OS curve of osteosarcoma patients in our study were subgrouped into either the metastatic or nonmetastatic groups. Significantly shorter OS was observed in patients with metastasis. Cox regression analysis was used to determine statistical significance. *P < 0.05, **P < 0.01, ns; no statistical significance. (L) Kaplan–Meier OS curve of osteosarcoma patients in our study subgrouped as having either low (staining score ≤ 1+) or high (staining score ≥ 2+) TOPK expression. Significantly shorter OS was observed in patients with high TOK expression. Cox regression analysis was used to determine statistical significance. *P < 0.05, **P < 0.01, ns; no statistical significance.

The immunostained slides were than underwent microscopic evaluation (Nikon Instruments Inc., Melville, NY, USA) and TOPK expression was categorized into four groups based on cytoplasmic staining intensity: 0, no staining, < 10% positive cells; 1+, weak staining, 10–25% positive cells; 2+, moderate staining, 26–50% positive cells; and 3+, strong staining, > 50% positive cells (Fig. 2A). The low TOPK expression subset comprised groups 0 and 1+, while groups 2+ and 3+ were defined as the high TOPK expression subset.

### 2.4 Human osteosarcoma cell lines

The human osteoblast cell line NHOST was acquired from Lonza Walkersville Inc. (Walkersville, MD, USA) and cultured in osteoblast growth medium (PromoCell, Heidelberg, Germany). The human osteosarcoma cell line KHOS was generously provided by Dr. Efstathios Gonos (Institute of Biological Research & Biotechnology, Athens, Greece) [27]. Other human osteosarcoma cell lines MG63, MNNGHOS, U2OS, and 143B were purchased from the American Type Culture Collection (ATCC) (Rockville, MD, USA). The recurrent human osteosarcoma cell line OSA1777 was established in our lab and previously authenticated by the ATCC database [28]. These cell lines were cultured in RPMI 1640 (GE Healthcare Life Sciences, Logan, UT, USA) supplemented with 10% fetal bovine serum (FBS, Sigma‐Aldrich, St. Louis, MO, USA) and 2% penicillin/streptomycin (Life Technologies, Carlsbad, CA, USA). All cell lines were cultured in a humidified 5% CO_2_ atmosphere at 37 °C.

### 2.5 Protein extraction and western blotting

The protein was extracted from cells and osteosarcoma tissue specimens by 1× RIPA lysis buffer (Sigma‐Aldrich) supplemented with protease inhibitor cocktail tablets (Roche Applied Science, Indianapolis, IN, USA). The concentrations of protein lysate were then evaluated by DC™ protein assay reagents (Bio‐Rad, Hercules, CA, USA) and a spectrophotometer SPECTRA max 340PC (Molecular Devices, LLC, San Jose, CA, USA).

Western blotting was carried out as previously described [29]. In brief, equal amounts of protein were first separated on 4–12% Bis‐Tris gels (NuPAGE^®^, Life Technologies) before transferred to nitrocellulose membranes. The membranes were then incubated at 4 °C overnight after they were blocked in 5% nonfat milk for 1 h with the following specific primary antibodies, TOPK (1 : 500 dilution, Cell Signaling Technology), poly (ADP‐ribose) polymerase (PARP) (1 : 1000 dilution, Cell Signaling Technology), Mcl‐1 (1 : 1000 dilution, Santa Cruz Biotechnology, Dallas, TX, USA), Survivin (1 : 1000 dilution, Cell Signaling Technology), and β‐actin (1 : 1000 dilution, Sigma‐Aldrich). Afterward, the membranes were cleaned with TBST three separate times for 5 min, before incubated with Goat anti‐Rabbit IRDye^®^ 800CW (926–32211, 1 : 10 000 dilution) and Goat anti‐Mouse IRDye^®^ 680LT secondary antibody (926–68020, 1 : 10 000 dilution) (Li‐COR Biosciences, Lincoln, NE, USA) at room temperature for 1 h. After being washed with TBST another three times, the band detection was determined using Odyssey^®^ CLx equipment (LI‐COR Bioscience) and odyssey software 3.0. The quantity of β‐actin was accessed to control equal loading of proteins.

### 2.6 Immunofluorescence evaluation

TOPK expression in osteosarcoma cells was visualized by immunofluorescence assays. The KHOS and U2OS cells were grown for three days in 24‐well plates and fixed with 4% paraformaldehyde for 15 min before being permeabilized with 100% methanol and then blocked with 1% BSA. Immunostaining was carried out with TOPK (1 : 200 dilution, Cell Signaling Technology) and β‐actin (1 : 500 dilution, Sigma‐Aldrich) antibodies at 4 °C overnight. The cells were incubated for an additional 1 h with Alexa Fluor 488 (Green)‐conjugated goat anti‐rabbit antibody or Alexa Fluor 594 (red) goat anti‐mouse antibody (Li‐COR Biosciences, Carlsbad, CA, USA). Nucleus counterstaining was performed with 1 μg·mL^−1^ Hoechst 33342 (Life Technologies). Cell images were obtained using a Nikon Eclipse Ti‐U fluorescence microscope (Diagnostic Instruments Inc., Sterling Heights, MI, USA) equipped with a SPOT RT™ digital camera. Green color reflects TOPK expression, blue represents nucleus, and red represents cytoplasm.

### 2.7 Silence TOPK expression by siRNA transfection and MTT assay

Silence TOPK expression in osteosarcoma cells was performed via small interfering RNA (siRNA) transfection. KHOS and U2OS cells were grown at a density of 4 × 10^3^ cells per well in 96‐well plates or 2 × 10^5^ cells per well in 12‐well plates and transfected with increasing concentrations (0, 10, 30, or 60 nm) of TOPK siRNA (5′‐GACCAUAGUUUCUUGUUAA‐3′) (Sigma‐Aldrich) using the Lipofectamine RNAiMax reagent (Invitrogen, Carlsbad, CA, USA) according to manufacturer instructions. Nonspecific siRNA (SIC001, Sigma‐Aldrich) was used as a negative control. Three days following transfection with TOPK siRNA, the proteins of KHOS and U2OS cells were extracted for protein measurement by western blotting. Cellular proliferation was evaluated by conventional 3‐(4,5‐dimethylthiazol‐2‐yl)‐2,5‐diphenyl tetrazolium bromide (MTT) assays. At the end of the 5‐day treatment, 20 µL of MTT (5 mg·mL^−1^, Sigma‐Aldrich) was added to each well of the 96‐well culture plates. After incubating at 37 °C in a humidified 5% CO_2_ atmosphere for 4 h, the resulting formazan product was solubilized with 100 µL of acid isopropanol and the absorbance was measured at a 490 nm wavelength on the SpectraMax Microplate^®^ Spectrophotometer (Molecular Devices LLC, Sunnyvale, CA, USA).

### 2.8 TOPK suppression by OTS514 inhibitor and MTT assay

The selective and potent TOPK inhibitor OTS514 ((R)‐9‐(4‐(1‐aminopropan‐2‐yl)phenyl)‐8‐hydroxy‐6‐methylthieno(2,3‐c)quinoline‐4(5H)‐one, Selleckchem, Houston, TX, USA) has been proven to inhibit the effects of TOPK in cancers such as lung cancer, kidney cancer, ovarian cancer, and hematologic malignancies *in vitro* and *in vivo* [11, 16, 17, 18, 19]. OTS514 inhibited TOPK activity with a half‐maximal inhibitory concentration (IC50) value of 2.6 nm [20]. Here, KHOS and U2OS cells were seeded into 96‐well plates at a concentration of 4 × 10^3^ cells per well and incubated with increasing concentrations (0, 6.25, 12.5, 25, and 50 nm) of OTS514 for 2, 3, or 5 days prior to the following experiments. After OTS514 treatment for 5 days, KHOS and U2OS proliferation was investigated using MTT assays as previously mentioned. A Nikon microscope (Nikon Instruments Inc.) was used to examine the morphological changes of KHOS and U2OS cells after 3 and 5 days of OTS514 treatment.

The effect of OTS514 on the chemosensitivity of osteosarcoma cell lines was also investigated. KHOS and U2OS cells were cultured in 96‐well plates as previously described and incubated with different concentrations of doxorubicin and cisplatin. Then, 10, 20, and 30 nm of OTS514 were added to each well of the treatment groups. At the end of the 5‐day treatment, MTT assays were used to determine the cytotoxic effects in both cell lines as previously described. The synergistic effects of OTS514 were further evaluated by synergyfinder 2.0, a well‐established web application for multidrug combination synergy analysis [30] (https://synergyfinder.fimm.fi). The degree of combination synergy was evaluated using a zero interaction potency (ZIP) model [31], which defines drug interactions as either synergistic (synergy score > 10), additive (synergy score −10 to 10), or antagonistic (synergy score < −10) [30].

### 2.9 Clonogenic assay

Also called a colony formation assay, the clonogenic assay is a well‐established *in vitro* cell survival model that quantifies a single cell’s ability to grow into a colony [32]. A clonogenic assay can be used to study the effectiveness of specific agents on cell proliferation and survival. Osteosarcoma KHOS and U2OS cells were seeded at 400 cells per well in 12‐well plates and treated with OTS514 at different concentrations (0, 6.25, 12.5, 25 nm) and then incubated for 10 days at 37 °C. Afterward, colonies were fixed with methanol for 10 min and then washed three times with PBS before staining with 10% Giemsa stain (Sigma‐Aldrich) for 20 min. Finally, cells were washed with flowing water and allowed to dry. Images of the stained colonies were captured using a digital camera (Olympus, Tokyo, Japan).

### 2.10 Wound healing—cell migration assay

Wound healing assays were utilized to test cell migration activities. KHOS and U2OS cells were seeded into 6‐well plates at a density of 4 × 10^5^ cells per well and incubated at 37 °C overnight. A sterile 30 µL tip was then used to scrape two parallel lines within the adherent cell layer. Next, 10 nm of OTS514 was added and left to incubate for 72 h in a low‐serum medium containing 2% FBS. Pictures of wounds were captured using a microscope (Nikon Instruments Inc.) with NIS‐Elements platform after 0, 24, 48, and 72 h of OTS514 treatment. The distance between the two edges of the scratch at five different sites of each image was measured to represent the average wound width. The following formula was applied to calculate the cell migration distance: (wound width at 0‐hour time point – wound width at the observed time point)/2.

### 2.11 Three‐dimensional (3D) cell culture

3D cell culture is an artificial environment that allows *in vitro* tumor cells to interact with their surroundings and grow in all the directions they would *in vivo* [33]. In our experiment, we prepared the hydrogel 3D culture system according to manufacturer protocol (VitroGel 3D‐RGD, #TWG002, TheWell Bioscience, Township, NJ, USA). We began with 250 µL of a 2 × 10^4^ cells·mL^−1^ suspension of KHOS and U2OS mixed with the prepared hydrogel 3D culture suspension in 24‐well culture plates. The other 250 µL of RPMI 1640 supplemented with 10% FBS and 2% penicillin/streptomycin was added to cover the hydrogel. Following, 10 nm of OTS514 was immediately put into the mixture. Spheroid formation of the osteosarcoma cells without treatment was considered as the negative control. The culture plates were then incubated at 37 °C in a humidified 5% CO_2_ atmosphere. Medium was changed every 48 h to provide sufficient nutrients and to prevent an osmolarity shift in the system. Images of spheroids were taken under the microscope every other day with NIS‐Elements platform (Nikon Instruments Inc.). At 10 days, the spheroid pictures were also taken on a Nikon Eclipse Ti‐U inverted fluorescence microscope (Nikon Instruments Inc.) after 15 min of incubation with 0.25 µm Calcein AM (Life Technologies).

### 2.12 Statistical analysis

graphpad prism 8 software (GraphPad Software, San Diego, CA, USA) and SPSS 23.0 (IBM Corp., Armonk, NY, USA) were utilized for statistical analyses. Nonparametric testing (Mann–Whitney *U*‐test) was utilized to compare and determine statistical significance of two independent groups. A one‐way analysis of variance (ANOVA) was utilized for multiple comparisons. The survival analyses were performed by Kaplan–Meier models. The correlations between different clinical characteristics and overall survival (OS) or recurrence‐free survival (RFS) was determined by Cox regression analysis. Only those parameters that were statistically significant (*P* < 0.05) in the univariate analysis were included in the multivariate analysis. The median OS, RFS, and hazard ratio (HR) were reported with a 95% confident interval (CI). A *P* value < 0.05 was considered statistically significant.

## 3 Results

### 3.1 TOPK gene is overexpressed in osteosarcoma

We first assessed TOPK gene expression from public databases, which included more than 68 000 samples and 72 different paired tissues from the GPL96 platform (HG‐U133A) of Affymetrix mRNA gene array GENT2 database [22, 34]. TOPK expression was significantly higher in cancerous samples compared with their normal tissue counterparts (*P* < 0.001). These included cancers of the breast, ovary, endometrium, cervix, colon, esophagus, stomach, kidney, liver, pharynx, thyroid, bone, and soft tissue sarcoma (Fig. 1A).

TOPK was significantly overexpressed in osteosarcoma within the RNA sequencing database. The osteosarcoma expression profile of TOPK mRNA was available from 88 samples from TARGET‐OS, six osteosarcoma cell lines from CCLE, and 396 normal bone or muscle tissues from GTEx. TOPK mRNA was significantly elevated in osteosarcoma tissue samples (40.8 ± 27.0 TPM, *P* < 0.001) and cell lines (101.0 ± 68.7 TPM, *P* < 0.001) compared with normal tissues (0.13 ± 0.22 TPM) (Fig. 1B). In addition, we found that TOPK gene expression significantly correlated with disease recurrence (*P* = 0.003) but not clinical characteristics such as age, gender, chemosensitivity, metastasis, or death (Fig. S1).

Furthermore, a survival analysis of TARGET‐OS data revealed a three‐year OS rate of 65.1% (median OS = 89.8 months (73.6–106.0)) in the high TOPK expression group, whereas the low expression group had a three‐year OS rate of 90.9% (median OS = 121.4 months (105.3–131.5)) (*P* = 0.042) (Fig. 1C). RFS showed no significant difference (Fig. 1D).

### 3.2 TOPK was highly expressed in our human osteosarcoma TMA, cell lines, and fresh tissues

To further support our findings, we evaluated TOPK expression in an osteosarcoma TMA. Of these patient tissues, 65 of 66 (98.5%) showed positive TOPK expression within the cytoplasm, ranging from staining group 0 (1 of 66, 1.51%); 1+ staining (10 of 66, 15.15%); 2+ staining (24 of 66, 36.36%), and 3+ staining (31 of 66, 46.98%) (Fig. 2A,B). Then, these stained specimens were divided into two categories: 0 and 1+ were defined as the low TOPK expression group (16.67%), whereas the 2+ and 3+ staining groups as being the high TOPK expression group (83.33%) (Fig. 2C, Table 1).

We also revealed elevated TOPK expression in osteosarcoma cell lines via western blot, including KHOS, MG63, MNNGHOS, OSA1777, U2OS, and 143B, with the human osteoblast cell line NHOST serving as negative control (Fig. 2D,E). We also investigated TOPK expression in fresh human osteosarcoma tissue specimens and found high expression in seven of the eight samples (87.5%) (Fig. 2F,G). We also localized TOPK expression via immunofluorescence of KHOS and U2OS and detected that TOPK protein was located primarily within the cytoplasm (Fig. S2). This result was consistent with our osteosarcoma TMA findings, which showed high TOPK expression in cytoplasmic localization within osteosarcoma tissues.

### 3.3 TOPK expression correlates with osteosarcoma clinical characteristics and prognosis

Based on the overexpression of TOPK in our osteosarcoma TMA, we analyzed whether TOPK expression correlates with patient clinical characteristics and prognosis. Higher TOPK expression was significantly associated with metastatic or recurrent osteosarcoma compared with primary disease alone (*P* = 0.009 and *P* = 0.03, respectively) (Fig. 2H). Additionally, the TOPK staining score was significantly higher in the osteosarcoma tissues from patients who later developed metastatic disease than those who did not (*P* = 0.008) (Fig. 2I). Moreover, the osteosarcoma tissues from nonsurvival patients showed significantly higher TOPK staining score than those patients who survived (*P* = 0.008) (Fig. 2J). Although a higher immunostaining score was observed among osteosarcoma tissues from those with recurrent, high‐grade, or chemoresistant (< 90% tumor necrosis) groups, statistical significance was not met. Expression of TOPK significantly correlated with metastasis and disease status (*P* = 0.039 and *P* = 0.046, respectively) but not other clinical parameters such as patient age, gender, tumor location, or histologic grade (Table 1).

Next, we performed an OS analysis to determine the prognostic value of TOPK expression in osteosarcoma. In our TMA analysis, the OS was 60.34% at three years, 55.60% at 5 years, and 45.51% at ten years. The median OS was 91.0 months (22.7–159.3 months). The OS of the high TOPK expression group was 54.55% at 3 years, 48.96% at 5 years, and 39.20% at ten years, with median OS at 65.40 months. In contrast, the OS of the low TOPK expression group was 90.48% at 3 and 5 years, and 79.17% at 10 years, with a median OS of 252.00 months (Table 2). A univariate analysis demonstrated metastasis (HR = 11.12 (2.68–46.19), *P* = 0.001) and TOPK expression (HR = 3.33 (1.03–10.82), *P* = 0.045) were poor prognostic predictors for OS (Table 2) (Fig. 2K,L). However, our multivariate analysis revealed only metastasis was an independent risk factor of OS for osteosarcoma patients.

**Table 2:** Univariate and multivariate overall survival analysis of prognostic factors in osteosarcoma.

Variable	Overall survival (%)			Median overall survival (months)	Univariate analysis		Multivariate analysis	
	3‐year	5‐year	10‐year		HR (95% CI)	*P* value	HR (95% CI)	*P* value
Age (years)								
< 20	60.87	51.66	41.33	85.93	1.05 (0.62–1.78)	0.869		
20–60	57.89	57.89	49.62	95.18				
> 20	75.00	50.00	25.00	84.00				
Gender								
Male	54.50	51.84	43.66	80.11	1.33 (0.70–2.53)	0.380		
Female	69.23	61.32	48.18	94.34				
Tumor site								
Femur	56.25	53.13	40.63	87.00	0.85 (0.67–1.07)	0.164		
Tibia	54.55	44.63	31.88	53.50				
Humerus	75.00	75.00	75.00	196.00				
Pelvis and vertebrae	31.41	31.41	31.41	35.14				
Others	100.00	85.71	68.57	132.00				
Histologic grade								
Low grade	88.89	88.89	88.89	171.75	2.82 (0.87–9.15)	0.085		
High grade	55.81	50.29	38.71	73.83				
Recurrence								
Absent	65.66	63.27	50.14	168.30	1.79 (0.96–3.34)	0.068		
Present	50.00	40.91	36.36	48.00				
Metastasis								
Absent	89.16	89.16	89.16	264.00	11.12 (2.68–46.19)	0.001*	10.45 (2.51–43.46)	0.001*
Present	48.94	42.55	29.16	46.50				
TOPK								
Low expression	90.48	90.48	79.17	252.00	3.33 (1.03–10.82)	0.045*	2.86 (0.87–9.43)	0.084
High expression	54.55	48.96	39.20	65.40				

CI, confident interval; HR, hazard ratio.

* Statistical significance (*P* < 0.05).

As a tertiary hospital and referral center, our samples included more advanced osteosarcoma patients, and therefore, more metastatic osteosarcomas were present in our TMA. To increase external validity to the general osteosarcoma population, our TMA was further analyzed in cases of osteosarcoma tissues from patients with only localized disease. After the exclusion of patients with metastasis at presentation, 53 osteosarcoma samples including 31 (58.5%) males and 22 (41.5%) females were analyzed. Among these patients, TOPK overexpression was significantly correlated with disease status, similar to the previous analysis, while no statistical significance with metastasis was observed (Table S1). The OS analysis revealed a 5‐year survival rate at 59.74% with a median OS of 137.07 months (45.9–230.1 months). The OS of the high TOPK expression group was 58.14% at 3 years, 50.97% at 5 years, and 43.69% at 10 years, with a median OS of 76.78 months. In contrast, the OS of the low TOPK expression group was 100% at 3 and 5 years and 87.5% at 10 years, with a median OS of 252.00 months (Table S2). In our univariate analysis, TOPK overexpression, metastasis, and recurrence were poor prognostic factors for OS, similar to the previous analysis. However, multivariate analysis revealed only metastasis was an independent risk factor (Table S2). In cases of localized osteosarcoma, presenting variables upon diagnosis are those which inform initial therapeutic decisions and therefore do not include subsequent variables such as recurrence or metastasis. Interestingly, TOPK expression has independent prognostic value for OS when only baseline variables are taken into consideration (except for subsequent recurrence and metastasis), with a hazard ratio of 4.48 (1.07–18.85, 95% CI) (Table S2). However, since there was no sufficient information about time to progression such as recurrence or metastasis, we cannot confirm TOPK as prognostic predictor for progression‐free survival at this time. Further study of TOPK expression in tumor tissues derived from larger scale samples of osteosarcoma patients is needed.

### 3.4 TOPK knockdown by siRNA decreases osteosarcoma cell proliferation

**Fig. 3:**
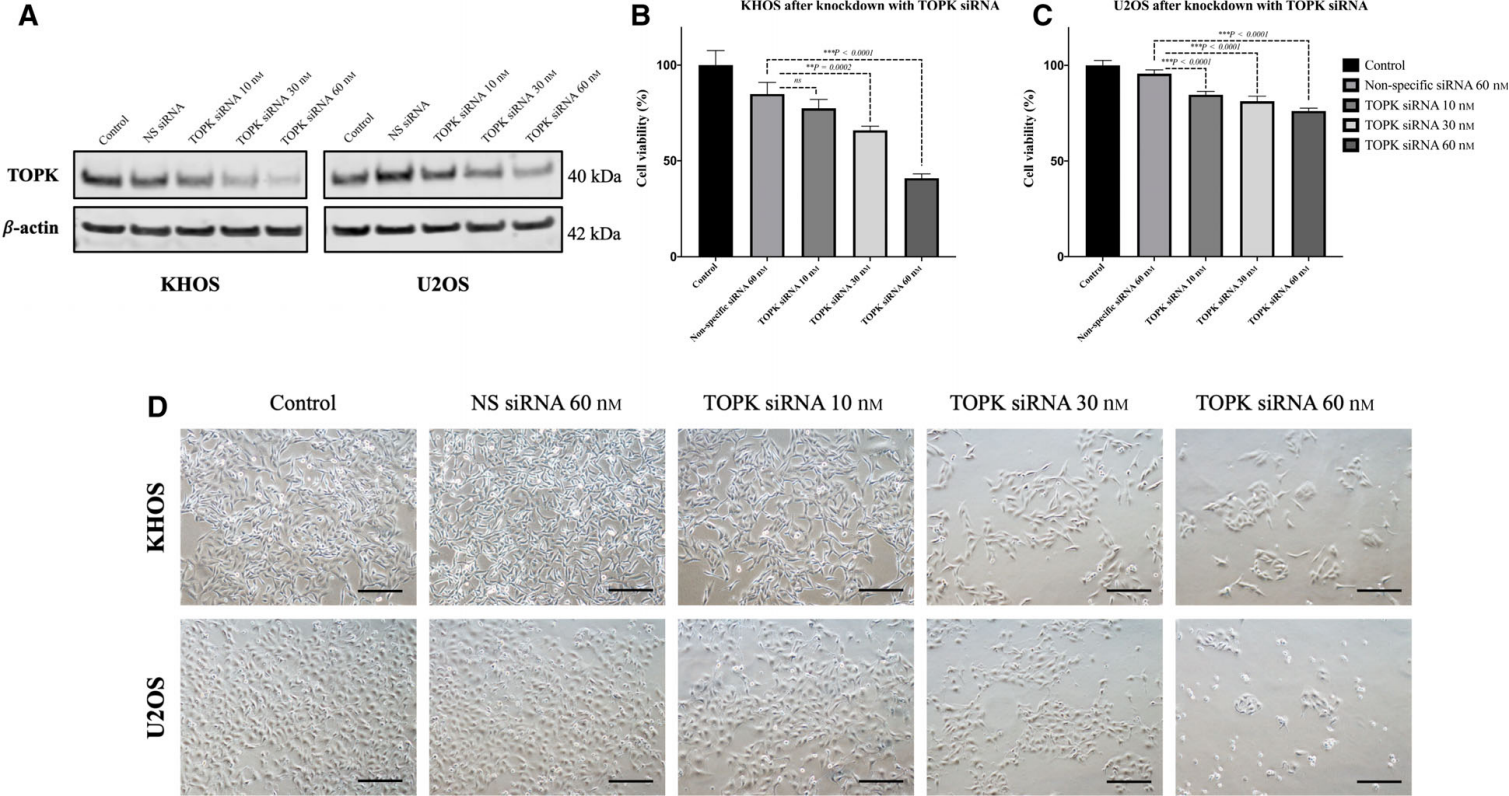
Effect of TOPK knockdown by specific siRNA on osteosarcoma cell line growth. (A) Western blot results display the expression of TOPK in KHOS and U2OS after transfection with different concentrations of TOPK‐specific siRNA. The data exhibit results of the independent triple experiment. (B) KHOS cell viability after transfected with TOPK‐specific siRNA. The data exhibit the mean ± SD of the experiment carried out in triplicate. Mann–Whitney *U*‐test was used to analyze statistical significance. ***P < 0.001. (C) U2OS Cell viability after transfected with TOPK‐specific siRNA. The data exhibit the mean ± SD of the experiment carried out in triplicate. Mann–Whitney *U*‐test was used to analyze statistical significance. ***P < 0.001. (D) Microscopic images demonstrate cell number reduction in both KHOS and U2OS cell lines after TOPK knockdown with specific siRNA for 5 days. (Scale bar; 100 µm). ***P < 0.001, ns; no statistical significance.

To investigate the function of TOPK in osteosarcoma growth and proliferation, we knocked down its expression via TOPK siRNA and quantified the subsequent changes within cell lines. We first used immunofluorescent assays and western blots to assess TOPK expression in osteosarcoma cell lines after TOPK siRNA transfection. Immunofluorescent study unveiled a marked decrease in TOPK fluorescence in both KHOS and U2OS following 60 nm of TOPK siRNA transfection (Fig. S2). Western blots further confirmed a notable decrease in TOPK expression with increasing concentrations of siRNA in KHOS and U2OS. This effect was absent in cells transfected with nonspecific siRNA (Fig. 3A).

In MTT assays, osteosarcoma cell viability was decreased in a dose‐dependent manner in KHOS and U2OS following transfection with increasing concentrations of TOPK siRNA over 5 days. Similar finding was not observed in the control groups, including untreated cells and the nonspecific siRNA‐transfected cells (Fig. 3B‐D).

### 3.5 Pharmacological TOPK inhibition with OTS514 in osteosarcoma cells

**Fig. 4:**
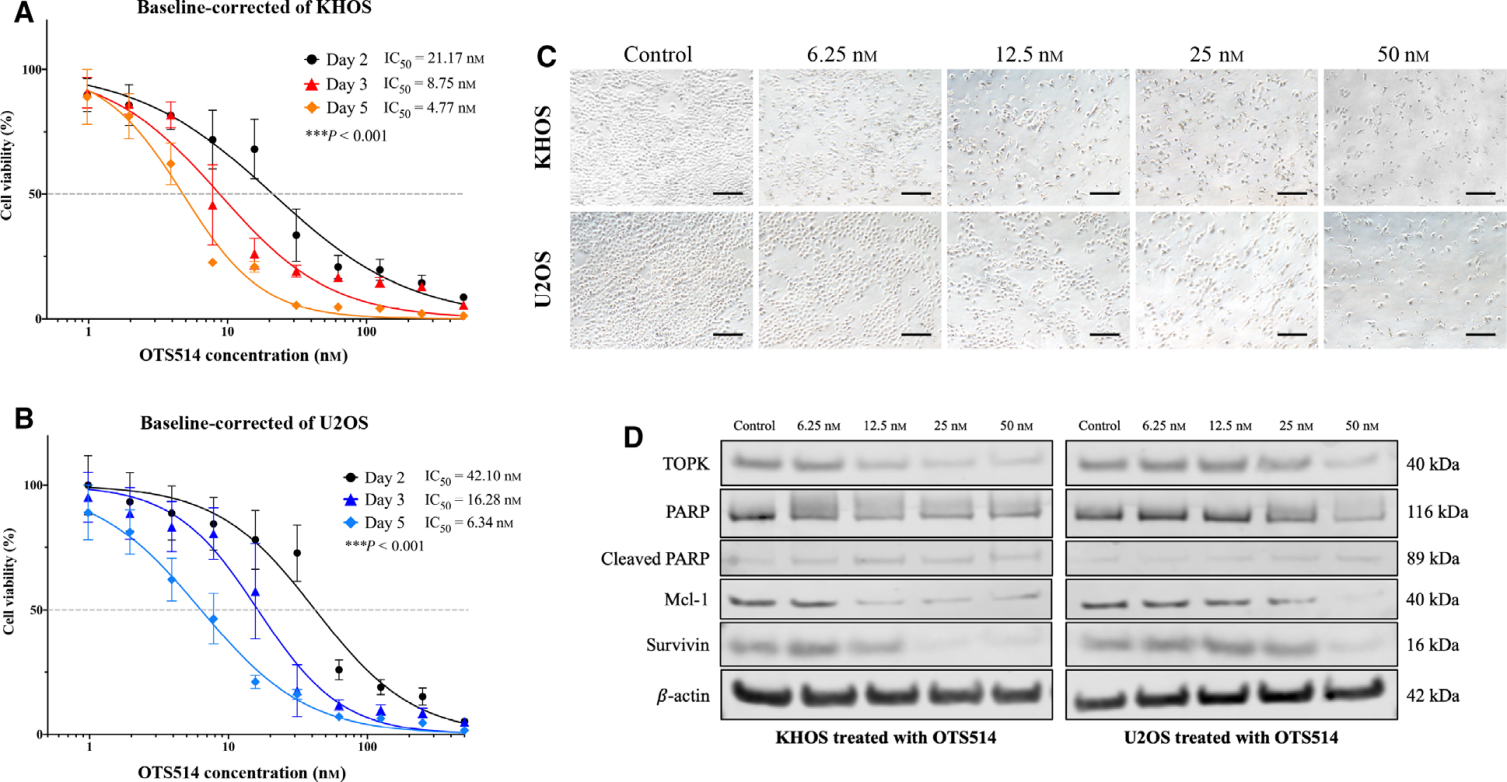
Effect of TOPK inhibition by OTS514 on osteosarcoma cells. (A) Dose–response curve of KHOS treated by OTS514 for 2, 3, and 5 days. The data exhibit mean ± SD of the independent triple experiment. ANOVA was used to analyze statistical significance. ***P < 0.001. (B) Dose–response curve of U2OS treated by OTS514 for 2, 3, and 5 days. The data exhibit mean ± SD of the independent triple experiment. ANOVA was used to analyze statistical significance. ***P < 0.001. (C) Microscopic pictures display cell number reduction after TOPK inhibition with OTS514 for 5 days in both KHOS and U2OS (Scale bar; 100 µm). (D) Expression of proteins likely involved in antiapoptotic activity of TOPK in KHOS and U2OS following treatment with OTS514 for 3 days, as examined via western blot.

We assessed whether these findings occurred with TOPK inhibition within KHOS and U2OS via the TOPK inhibitor OTS514. A dose‐ and time‐dependent decrease in osteosarcoma cell viability was observed in KHOS and U2OS, with IC50 values for 5 days of OTS514 treatment at 4.77–21.17 nm and 6.34–42.10 nm, respectively (Fig. 4A,B). Similarly, we observed a reduction in viable cells and morphologic changes with increasing concentrations of OTS514 in KHOS and U2OS after 3 days of treatment (Fig. 4C). Western blots demonstrated TOPK and antiapoptotic proteins Mcl‐1 and Survivin significantly decreased in a dose‐dependent manner, while apoptotic cleavage of PARP increased after incubation of KHOS and U2OS with 6.25, 12.5, 25, and 50 nm of OTS514 for 72 h (Fig. 4D). To exclude the confounding effect of DMSO, we also treated the osteosarcoma cell lines with different concentrations of DMSO and found no significant biological effect of DMSO to osteosarcoma cell growth and proliferation.

**Fig. 5:**
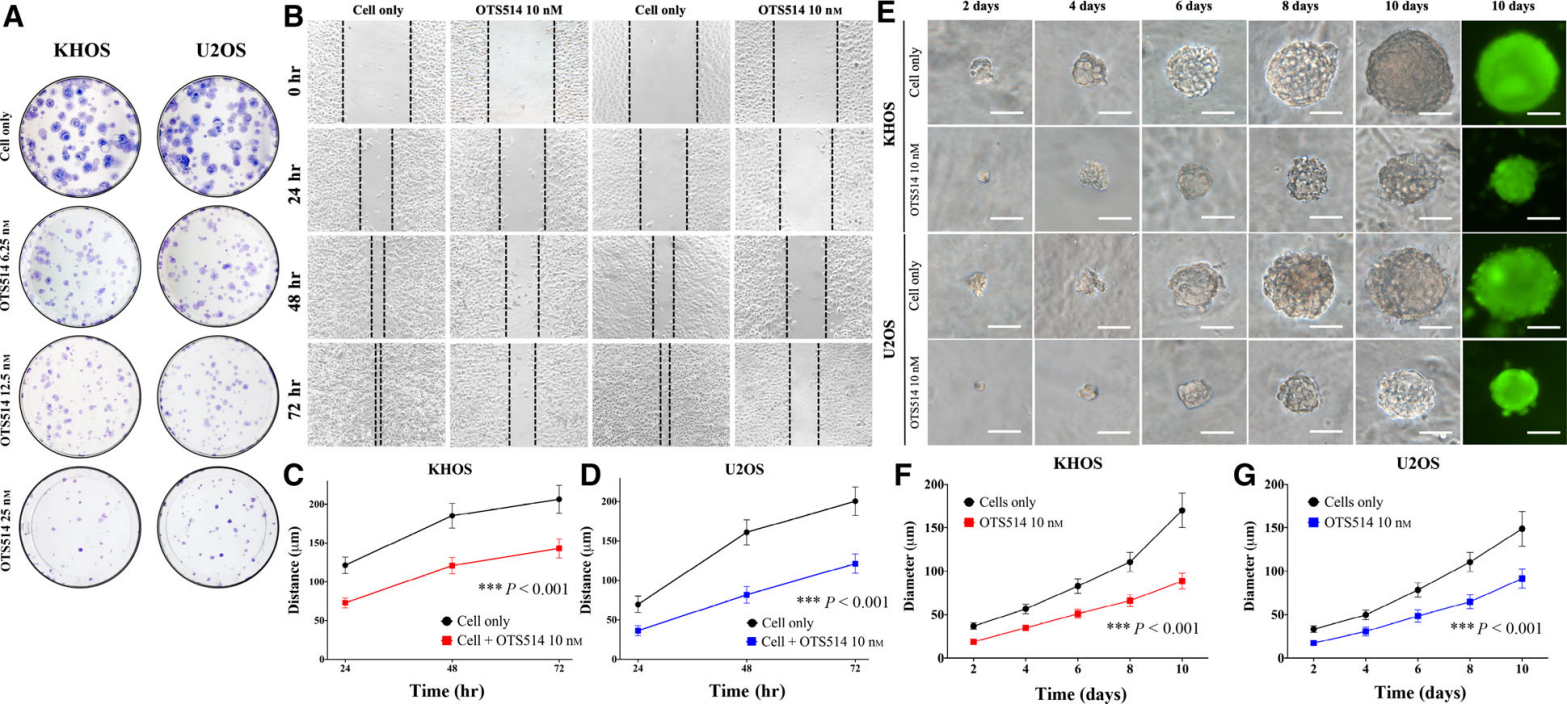
TOPK inhibition by OTS514 reduced osteosarcoma cell clonogenicity and migration *in vitro* and decreased spheroid diameter in 3D cell culture. (A) Images of colony formation in KHOS and U2OS. The numbers of colonies and their sizes were markedly decreased in osteosarcoma cells treated with OTS514. The data exhibit results of the independent triple experiment. (B) Representative images of KHOS and U2OS migration after 10 nm of OTS514 treatment for 24, 48, and 72 h. The data exhibit results of the independent triple experiment. (C) The curve of cell migration distance of KHOS treated with OTS514 showed significant decrease compared with untreated cells. The data exhibit mean ± SD of the independent triple experiment. Mann–Whitney *U*‐test was used to analyze statistical significance. ***P < 0.001. (D) The curve of cell migration distance of U2OS cells treated with OTS514 showed significant decrease compared with untreated cells. The data exhibit mean ± SD of the independent triple experiment. Mann–Whitney *U*‐test was used to analyze statistical significance. ***P < 0.001. (E) Images of spheroid formation of KHOS and U2OS cultured with 10 nm of OTS514. Fluorescence images were taken after 10 days of culturing. Spheroid formation of KHOS and U2OS cultured with OTS514 was significantly smaller than untreated cells at all observation points. The data show results of the independent triple experiment. (Scale bar; 50 µm). (F) The curve of spheroid diameter of KHOS treated with OTS514 showed significant decrease compared with untreated cells. The data show mean ± SD of the independent triple experiment. Mann–Whitney *U*‐test was used to analyze statistical significance. ***P < 0.001. (G) The curve of spheroid diameter of U2OS treated with OTS514 showed significant decrease compared with untreated cells. The data show mean ± SD of the independent triple experiment. Mann–Whitney *U*‐test was used to analyze statistical significance. ***P < 0.001.

We next investigated the effect of OTS514 on osteosarcoma cell colony formation within a clonogenic assay. KHOS and U2OS showed a dose‐dependent reduction in colony formation with OTS514 treatment compared with the untreated cells (Fig. 5A). Furthermore, as cell migration and invasion are hallmark features of metastasis and the primary cause of osteosarcoma patient death, we began a second set of experiments. After confirming in our TMA that TOPK expression significantly correlated with osteosarcoma metastasis, we exposed our cell lines with 10 nm of OTS514 to reveal the effect of TOPK inhibition on *in vitro* osteosarcoma cell migration. This resulted in a significant time‐dependent decrease in cell migration in both KHOS and U2OS (Fig. 5B‐D).

Additionally, we examined whether suppression via OTS514 would affect spheroid formation in a 3D cell culture. The spheroid diameters in OTS514‐treated KHOS and U2OS were significantly smaller than the untreated cells (Fig. 5E‐G). After 10 days of 10 nm OTS514 treatment, the spheroid diameters of KHOS were 52.1% of the untreated KHOS group (*P* < 0.001, Fig. 5F). A similar result was also observed in U2OS, where the spheroid diameters were 61.5% of the untreated U2OS group (*P* < 0.001, Fig. 5G).

### 3.6 TOPK inhibition promotes chemosensitivity in osteosarcoma cells

**Fig. 6:**
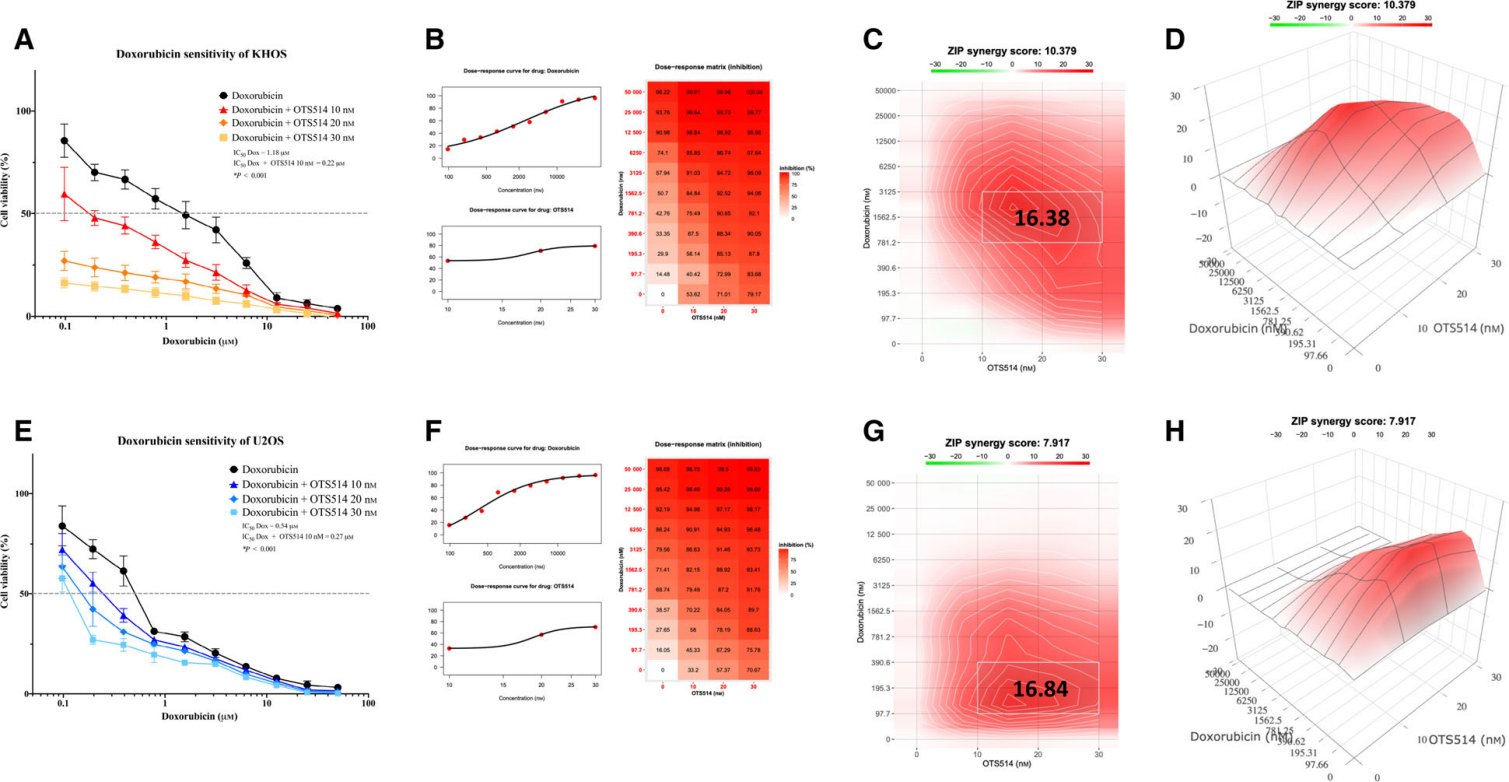
TOPK inhibitor synergy with doxorubicin in osteosarcoma cells. (A) Dose–response curve of doxorubicin sensitivity in KHOS treated with different concentrations of OTS514. Cell viability significantly decreased with increasing concentrations of OTS514. The data show mean ± SD of the independent triple experiment. Mann–Whitney *U*‐test was used to analyze statistical significance. ***P < 0.001. (B) Dose–response curves and matrix of doxorubicin combined with OTS514 in KHOS, as analyzed by synergyfinder 2.0. The data show results of the independent triple experiment. (C) Two‐dimensional synergy map showing synergistic effects of OTS514 combined with doxorubicin in KHOS with Zero Interaction potency (ZIP) score: 10.379. The most synergistic area in the interaction map was 10–30 nm of OTS514 and 781.2–3125 nm of doxorubicin, with ZIP score: 16.38. (D) Three‐dimensional synergy illustration depicted the result from Fig. 6C. (E) Dose–response curve of doxorubicin sensitivity in U2OS treated with different concentrations of OTS514. Cell viability significantly decreased with increasing concentrations of OTS514. The data show mean ± SD of the independent triple experiment. Mann–Whitney *U*‐test was used to analyze statistical significance. ***P < 0.001. (F) Dose–response curves and matrix of doxorubicin combined with OTS514 in U2OS, as analyzed by synergyfinder 2.0. The data show results of the independent triple experiment. (G) Two‐dimensional synergy map showing additive effect of OTS514 combined with doxorubicin in U2OS with Zero Interaction potency (ZIP) score: 7.917. The most synergistic area in the interaction map was 10–30 nm of OTS514 and 97.7–390.6 nm of doxorubicin, with ZIP score: 16.84. (H) Three‐dimensional synergy illustration depicted the result from Fig. 6G.

TOPK inhibitors have known anticancer effects when combined with additional chemotherapy or radiation therapy [9]. As doxorubicin and cisplatin are among the most popular chemotherapeutics in osteosarcoma [3, 35], we sought to investigate whether additional TOPK inhibition would enhance osteosarcoma chemosensitivity. MTT assays were used to compare viabilities of KHOS and U2OS treated with combinations of increasing concentrations of doxorubicin or cisplatin alongside the TOPK inhibitor OTS514. We found increasing concentrations of OTS514 decreased the IC50 of doxorubicin in both KHOS and U2OS in a dose‐dependent manner (Fig. 6). A synergistic analysis revealed OTS514 did result in a synergistic anticancer effect on KHOS (ZIP synergy score = 10.379) (Fig. 6A–D); however, the same combination had an additive effect in U2OS (ZIP synergy score = 7.917) (Fig. 6E‐H). Conversely, OTS514 therapy produced an additive anticancer effect alongside cisplatin in KHOS (ZIP synergy score = 9.945) and a synergistic effect in U2OS (ZIP synergy score = 11.336) (Fig. S3).

## 4 Discussion

TOPK has recently become a potential therapeutic target in cancer as it has shown overexpression within various tumors compared with their normal counterparts [11, 14, 15, 16, 17]. This observation has been supported by the recent expansion of cancer genome databases and has made the selection of cancer targets such as TOPK a higher‐yield process for preclinical research [22, 36, 37, 38, 39]. In our present study, analysis of the GENT2 database revealed a notably higher TOPK expression within bone tumors and the TARGET‐OS RNA sequencing database showed a significantly higher TOPK mRNA expression in osteosarcoma tissues compared with normal bone and muscle tissues in the GTEx database. Furthermore, TOPK mRNA was highly expressed in osteosarcoma cell lines from the CCLE repository compared with healthy tissues. These finding were clinically relevant, as a combined database analysis supported TOPK gene overexpression as a poor prognostic indicator for osteosarcoma patient survival.

Drawing from our promising bioinformatic analysis, we evaluated and validated TOPK expression within our own osteosarcoma tissues and cell lines. The TMA data showed 98.5% of osteosarcoma tissue samples expressed TOPK, of which a majority 83.3% had high expression (staining score ≥ 2+). Similarly, a high quantity of TOPK protein was detected in fresh osteosarcoma tissues, and expression was elevated in all tested osteosarcoma cell lines compared with normal osteoblast cells.

Recent studies have indicated TOPK expression is a poor prognostic factor in cancers of the lung, ovary, kidney, colon, as well as leukemia, glioblastoma, and melanoma [11, 14, 15, 16, 17, 18, 19, 39]. In our TMA analysis, higher TOPK expression associated with metastasis and shorter OS. Particularly, 42 of 47 (89.4%) of the tissue samples from the patients with metastatic disease had high TOPK expression. Additionally, osteosarcoma patients with elevated TOPK expression had shorter OS compared with those with low expression, with a HR of 3.33 by univariate analysis. These results were significant and consistent with our TOPK gene expression analysis from the TARGET‐OS and GENT2 public databases, and endorse the prognostic significance of TOPK expression in osteosarcoma.

Knockdown of TOPK using either siRNA or short hairpin RNA (shRNA) has been proven to decrease tumor cell proliferation and induce apoptosis in multiple cancers [10, 40, 41, 42, 43]. To verify the functional roles of TOPK in osteosarcoma growth and proliferation, we conducted a knockdown experiment using a TOPK‐specific siRNA. Accordingly, there was a significant reduction in cell viability and growth in KHOS and U2OS upon TOPK suppression.

Of the various TOPK inhibitors available such as OTS514, HI‐TOPK‐032, and ADA‐07, we elected to use OTS514 as it is the most potent and target specific [9, 11, 18]. Recently, preclinical studies including xenograft models have shown OTS514 effectively inhibits tumor growth and dissemination in a dose‐dependent manner for cancers such as lung cancer, kidney cancer, ovarian cancer, myeloma, and leukemia [11, 16, 17, 18, 19]. In our work, we performed *in vitro* TOPK loss‐of‐function studies to determine its significance in osteosarcoma cell proliferation and growth. Similarly, TOPK inhibition with OTS514 decreased KHOS and U2OS growth and proliferation in a dose‐ and time‐dependent manner. While the exact molecular mechanism of TOPK inhibition in osteosarcoma is unclear, we report a marked decrease in the antiapoptotic proteins Mcl‐1 and Survivin alongside increased apoptotic cleavage of PARP. Therefore, TOPK likely promotes proliferation through an inhibition of apoptosis. Previous studies have also found OTS514 to associate with FOXM1 and MELK in TOPK‐expressing tumors [11, 16, 17, 18]. Most recently, TOPK was shown to positively regulate TBX3 in the TGF‐β/Smad signaling pathway in breast cancer, hence enhancing epithelial–mesenchymal transition (EMT) and tumor cell invasion [42]. These studies in other cancers warrant additional work to assess whether these TOPK pathways exist in osteosarcoma, as they may highlight potent and combined targeted therapy selection.

Clonogenic assays quantify the ability of a single cancer cell to form colonies *in vitro* [44, 45]. We show the number and size of colonies from KHOS and U2OS were reduced in a dose‐dependent manner with increasing OTS514 (Fig. 5A). As TOPK was highly expressed in 89.4% of the tissues of osteosarcoma patients with metastatic disease, we further examined the role of TOPK within *in vitro* osteosarcoma cell migration. Cell migration significantly decreased in both KHOS and U2OS following treatment with OTS514 in a time‐dependent manner (*P* < 0.001, Fig. 5B‐D). It is therefore likely that TOPK contributes to osteosarcoma cell migration and eventual distant metastasis. This finding is consistent with results in colon cancer, where TOPK regulates p53‐ and Akt‐mediated migration and metastasis to mouse liver tissue [46]. TOPK also promotes cancer stem cell self‐renewal, migration, and metastasis in neuroblastoma [21]. Taken together, our results demonstrate the importance of TOPK in metastasis and the ability of OTS514 to mitigate this effect in osteosarcoma. This is especially important clinically because pulmonary metastasis remains the primary mode of osteosarcoma patient mortality, and currently used chemotherapeutics have limited benefit in cases of tumor dissemination.

Given 3D cell culture is an approved *in vitro* model of the *in vivo* environment [33, 47], we sought to validate the effects of OTS514 on osteosarcoma cell proliferation within this medium. We show the diameters of osteosarcoma spheroid treated with OTS514 are notably decreased compared with untreated cells (*P* < 0.001, Fig. 5E–G). Previous studies have also demonstrated the reduction in *in vivo* tumor growth and dissemination in mouse models following OTS514 treatment [11]. Finally, we demonstrate OTS514 inhibits osteosarcoma growth synergistically when used alongside doxorubicin and cisplatin in both KHOS and U2OS. Our work show TOPK is a promising biomarker and therapeutic target for osteosarcoma treatment, particularly when administered in combination with standard osteosarcoma therapeutics.

Previous studies suggest that TOPK plays a role in cell cycle regulation and mitotic progression. Moreover, TOPK expression is minimal in differentiated cells, whereas its overexpression is a pathophysiological feature of various malignancies. Therefore, a specific TOPK inhibitor may have anticancer activity while minimizing off‐target toxicity. Our findings similarly suggest that a specific TOPK inhibitor may have therapeutic roles in osteosarcoma treatment; however, its clinical application for osteosarcoma is complex. Because of its genomic heterogenicity, no single specific genomic and molecular target in osteosarcoma tumorigenesis has been identified. This study provides promising new data into the molecular biology of osteosarcoma, but further investigation into the molecular mechanisms behind TOPK in osteosarcoma is needed.

## 5 Conclusions

In summary, our study shows that TOPK is aberrantly expressed in osteosarcoma and significantly associates with shorter OS. Therapeutically, TOPK inhibition decreases osteosarcoma cell growth, proliferation, migration, and dissemination. Of note, application of a potent and specific TOPK inhibitor has synergistic effects alongside the commonly used osteosarcoma therapeutics cisplatin and doxorubicin. These results support TOPK as a prognostic predictor for OS and potential target in osteosarcoma treatment that warrants further mechanistic and *in vivo* investigation.

## Conflict of interest

The authors declare no conflict of interest.

## Author contributions

PT and ZD conceptualized and designed the study. PT and RW performed experiments. PT, RW, and ZD contributed to acquisition of data. PT, RW, AS, NF, SD, FH, and ZD analyzed and interpreted the data. PT, DD, AS, NF, SD, FH, and ZD contributed to writing, review, and revision of the manuscript.

### Peer Review

The peer review history for this article is available at https://publons.com/publon/10.1002/1878‐0261.13039.

## Supporting information


**Fig. S1.** Correlations between TOPK gene expression and clinicopathology in the TARGET‐OS database. (A) Table and (B) bar charts representing correlations between TOPK mRNA expression and different clinical parameters in osteosarcoma patients retrieved from the Therapeutically Applicable Research to Generate Effective Treatments on Osteosarcoma (TARGET‐OS) database.

**Fig. S2.** TOPK expression in osteosarcoma cells by immunofluorescence. Expression of TOPK in osteosarcoma cell lines, including only cells and transfection with nonspecific siRNA (60nM) or TOPK siRNA (60 nM). Immunofluorescence signals include TOPK (green), β‐actin (red in cytoplasm), and Hoechst 33342 (blue in nuclei). The green fluorescence signal illustrating TOPK protein was localized in the cytoplasm of osteosarcoma cells and was apparently inhibited by TOPK siRNA. (Scale bar; 50 µm).

**Fig. S3.** TOPK inhibitor synergy with cisplatin in osteosarcoma cells. (A) Dose–response curve of cisplatin sensitivity in KHOS treated with different concentrations of OTS514. Cell viability was significantly decreased with increasing OTS514 concentrations. The data show mean ± SD of the independent triple experiment. (B) Dose–response curves and matrix of cisplatin combined with OTS514 in KHOS analyzed by SynergyFinder 2.0. (C) Two‐dimensional synergy map showing additive effect of OTS514 combined with cisplatin in KHOS, with Zero Interaction potency (ZIP) score: 9.945. The most synergistic area in the interaction map was 10 – 30 nM of OTS514 and 390.6 – 1562.5 nM of cisplatin, with ZIP score: 15.35. (D) Three‐dimensional synergy illustration depicting the result from Supplementary Figure 3C. (E) Dose–response curve of cisplatin sensitivity in U2OS treated with different concentrations of OTS514. Cell viability was significantly decreased with increasing concentrations of OTS514. The data show mean ± SD of the independent triple experiment. (F) Dose–response curves and matrix of cisplatin combined with OTS514 in U2OS, analyzed by SynergyFinder 2.0. (G) Two‐dimensional synergy map showing synergistic effect of OTS514 combined with cisplatin in U2OS cells with Zero Interaction potency (ZIP) score: 11.336. The most synergistic area in the interaction map was 10 – 30 nM of OTS514 and 781.2 – 3125 nM of cisplatin, with ZIP score: 17.82. (H) Three‐dimensional synergy illustration depicting the result from Supplementary Figure 3G. *** *p*<0.001.

**Table S1.** Correlations between TOPK expression and clinicopathology in patients with localized osteosarcoma.

**Table S2.** Univariate and multivariate OS analysis of prognostic factors in patients with localized osteosarcoma at diagnosis.

## Data Availability

The data that support the findings of this study are available from the corresponding author (zduan@mednet.ucla.edu) upon reasonable request.
