# Expression of Concern: T‐LAK cell‐originated protein kinase (TOPK): an emerging prognostic biomarker and therapeutic target in osteosarcoma

**DOI:** 10.1002/1878-0261.13711

**Published:** 2024-08-22

**Expression of Concern**: Thanindratarn P, Wei R, Dean DC, Singh A, Federman N, Nelson SD, Hornicek FJ and Duan Z (2021) T‐LAK cell‐originated protein kinase (TOPK): an emerging prognostic biomarker and therapeutic target in osteosarcoma. *Mol Oncol* 15(12), 3721–37. https://doi.org/10.1002/1878-0261.13039.

This Expression of Concern is for the above article, published online on 29 June 2021 in Wiley Online Library (wileyonlinelibrary.com), and has been published by agreement between the journal Editor‐in‐Chief, Kevin Ryan; FEBS Press; and John Wiley & Sons Ltd.

The Expression of Concern has been published due to concerns raised by a third party regarding the duplication of image panels of Fig. 2A within the figure and with another image in a previously published article by an overlapping group of authors. The authors were able to provide a compilation of the image panels but could not supply the original microscopy image files upon request. Further concerns were raised about the data and the error bars depicted in Fig. 5C, D, F and G. The authors were not able to provide the original raw data; therefore, the journal team could not verify the validity of these charts and could not exclude that the above‐mentioned concerns affect the overall conclusions of the article. Therefore, the journal has decided to issue an Expression of Concern to inform and alert the readers.

