# Supplementary material for: T‐LAK cell‐originated protein kinase (TOPK): an emerging prognostic biomarker and therapeutic target in osteosarcoma
Source: Mol Oncol. 2021 Jun 29;15(12):3721–37. doi: 10.1002/1878-0261.13039 (PMC8637563; doi:10.1002/1878-0261.13039)

(A)

Clinicopathological features		PBK/TOPK gene expression (TPM)	*p* value
Age	≥16 yrs	39.1±23.8	0.77
	<16 yrs	41.6±25.9	
Gender	Male	43.8±27.5	0.31
	Female	36.5±21.1	
Disease status	Non-metastatic	40.3±24.0	0.91
	Metastatic	41.6±28.3	
Chemotherapeutic response	Good	45.0±28.0	0.54
	Poor	40.8±27.9	
Death	Yes	47.3±25.7	0.084
	No	37.4±24.2	
Recurrence	Yes	49.2±26.2	0.003*
	No	33.2±21.6	

(B)

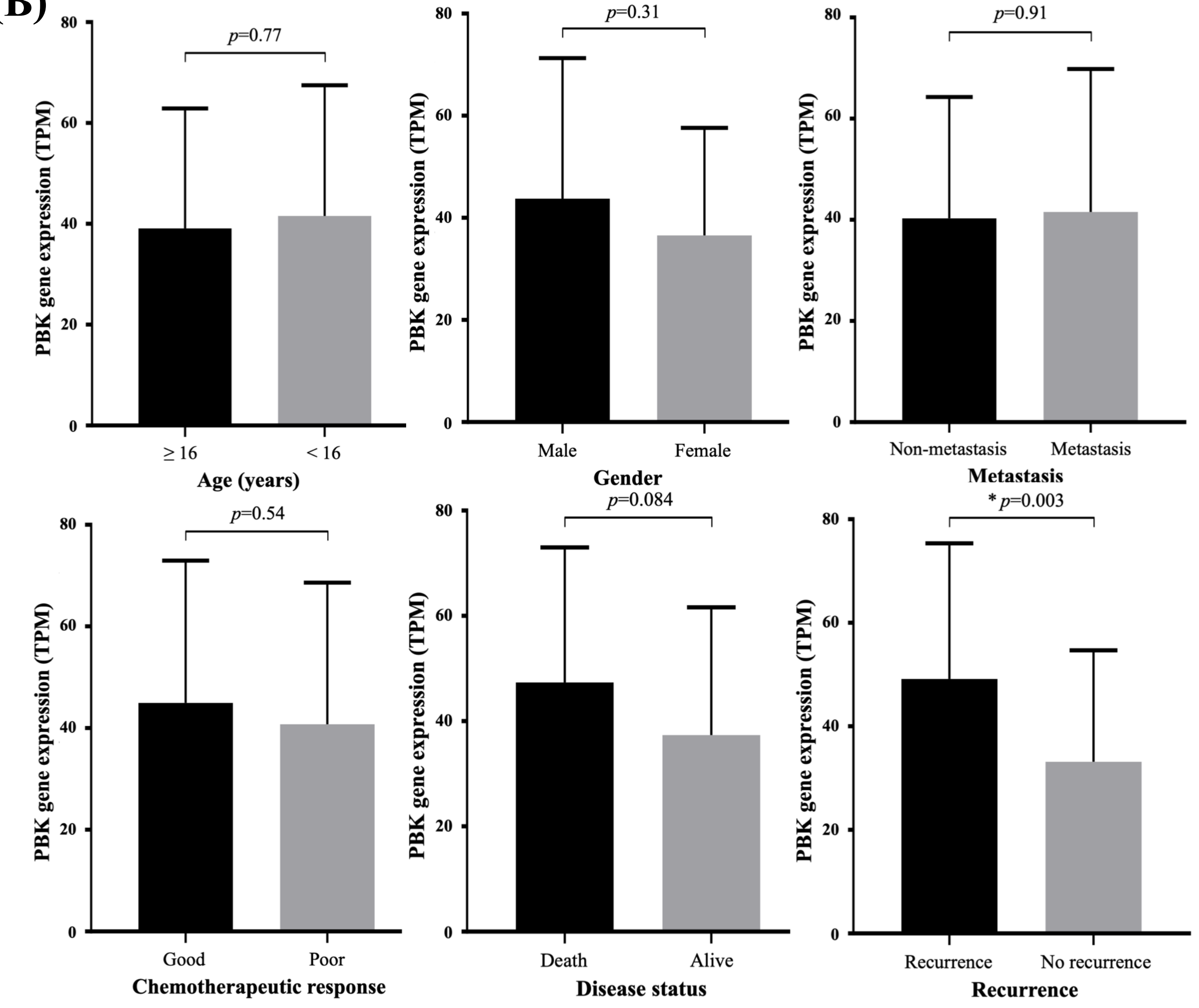

Supplement: Supplementary file 1 — Fig. S1. Correlations between TOPK gene expression and clinicopathology in the TARGET‐OS database. (A) Table and (B) bar charts representing correlations between TOPK mRNA expression and different clinical parameters in osteosarcoma patients retrieved from the Therapeutically Applicable Research to Generate Effective Treatments on Osteosarcoma (TARGET‐OS) database. [file MOL2-15-3721-s003.pdf]
