# Supplementary figures and images for: T‐LAK cell‐originated protein kinase (TOPK): an emerging prognostic biomarker and therapeutic target in osteosarcoma
Source: Mol Oncol. 2021 Jun 29;15(12):3721–37. doi: 10.1002/1878-0261.13039 (PMC8637563; doi:10.1002/1878-0261.13039)

# KHOS

Control

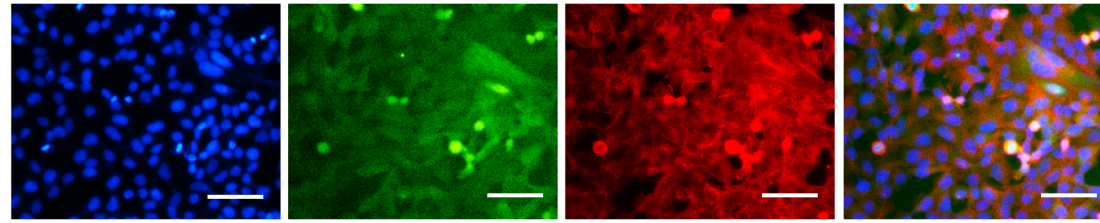

NS siRNA 60 nM

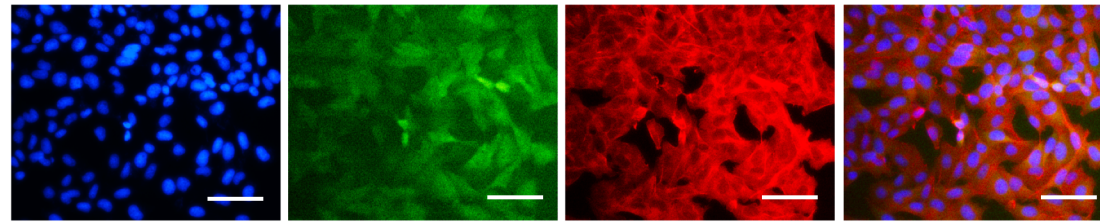

TOPK siRNA 60 nM

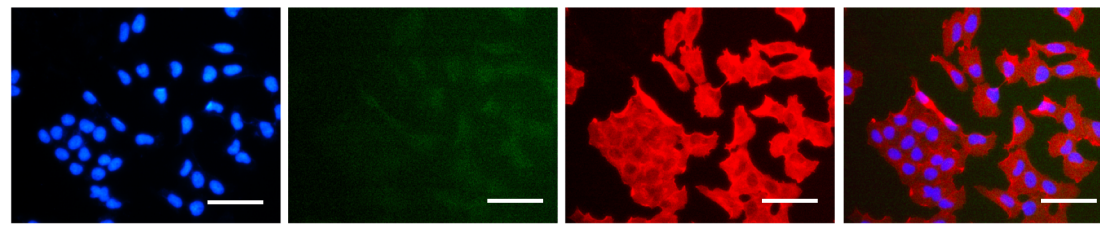

# U2OS

Control

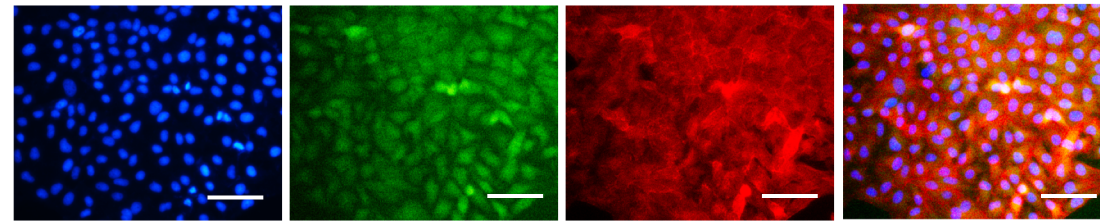

NS siRNA 60 nM

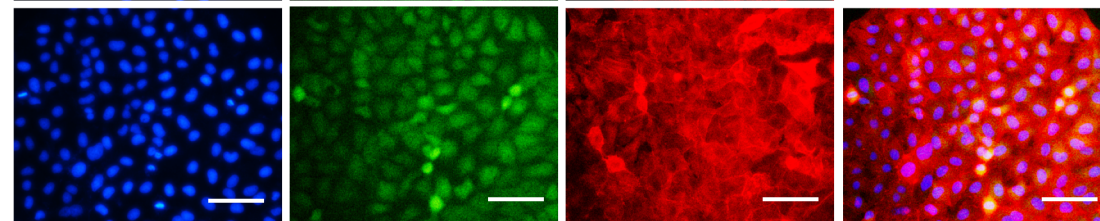

TOPK siRNA 60 nM

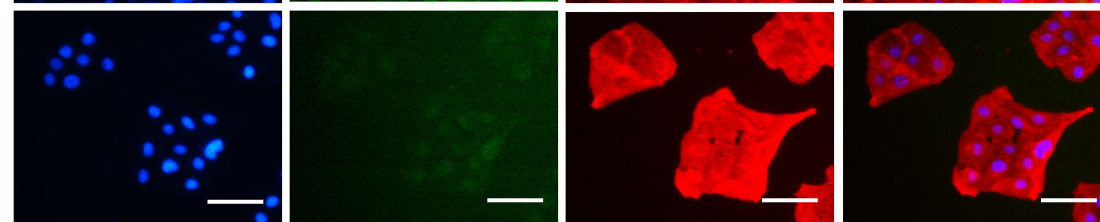

Supplement: Supplementary file 2 — Fig. S2. TOPK expression in osteosarcoma cells by immunofluorescence. Expression of TOPK in osteosarcoma cell lines, including only cells and transfection with nonspecific siRNA (60nM) or TOPK siRNA (60 nM). Immunofluorescence signals include TOPK (green), β‐actin (red in cytoplasm), and Hoechst 33342 (blue in nuclei). The green fluorescence signal illustrating TOPK protein was localized in the cytoplasm of osteosarcoma cells and was apparently inhibited by TOPK siRNA. (Scale bar; 50 µm). [file MOL2-15-3721-s002.pdf]

**(A)****Cisplatin sensitivity of KHOS**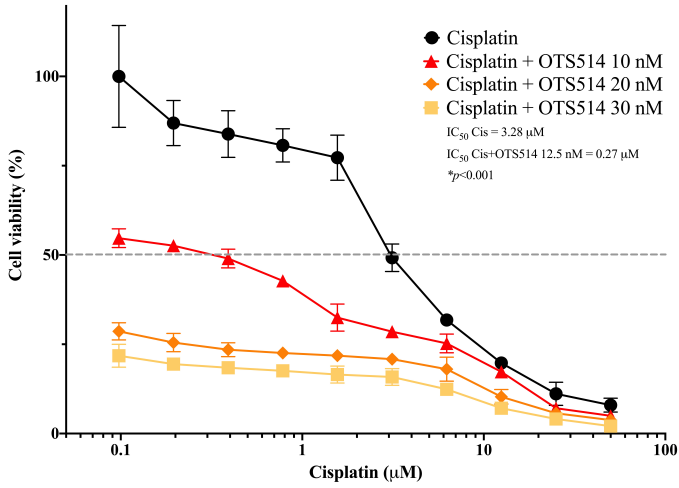**(B)**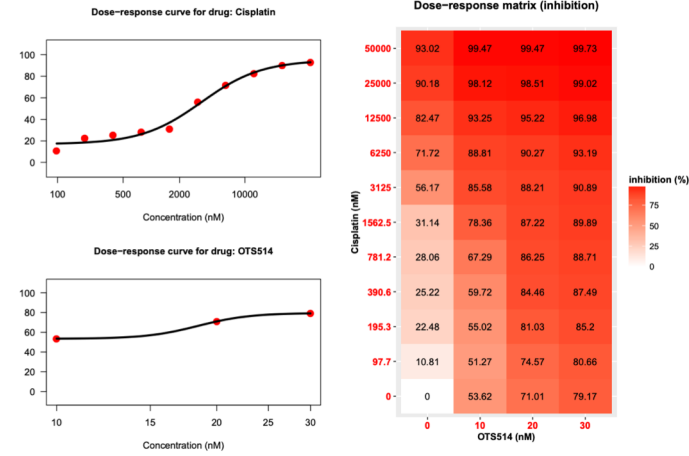**(C)**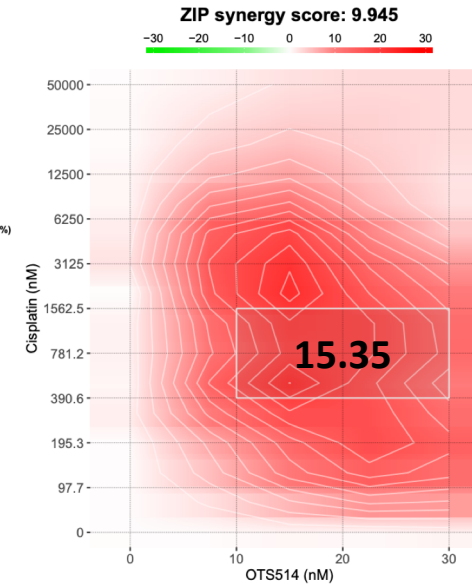**(D)**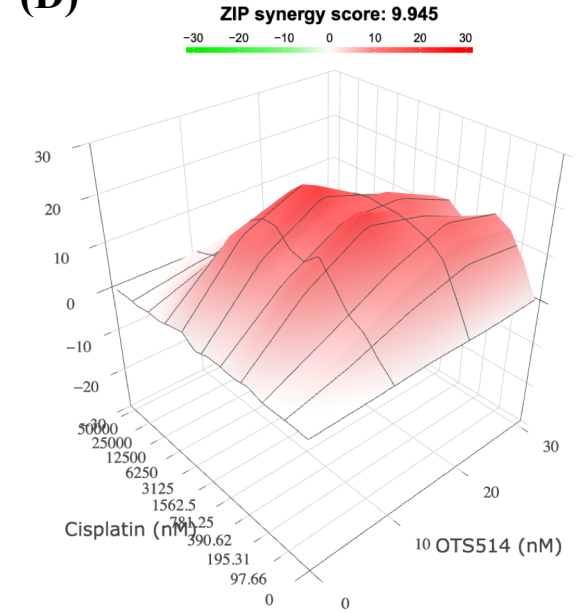**(E)****Cisplatin sensitivity of U2OS**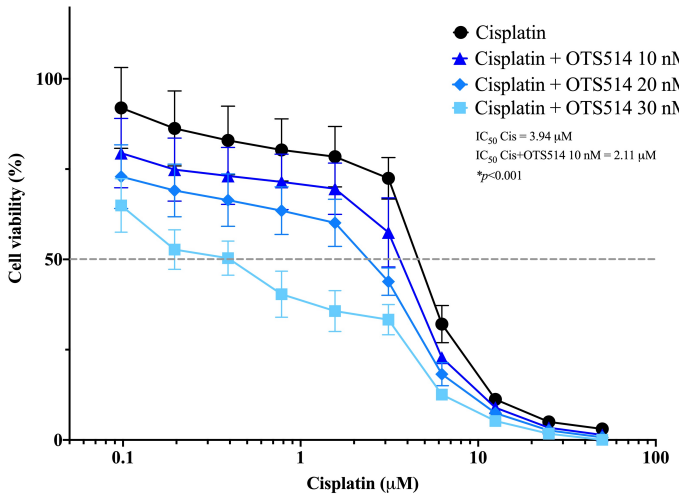**(F)**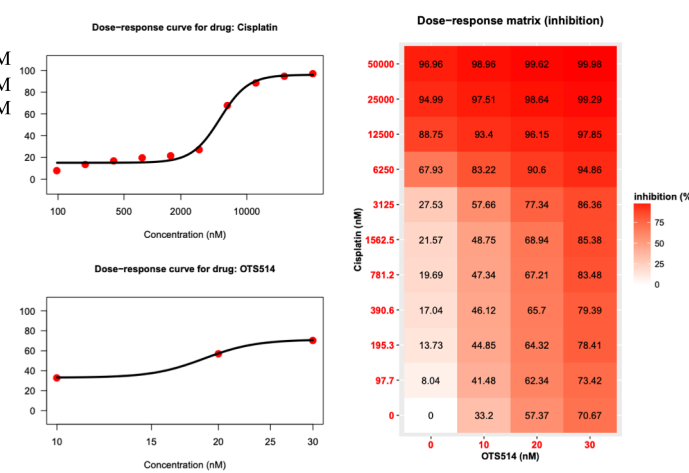**(G)**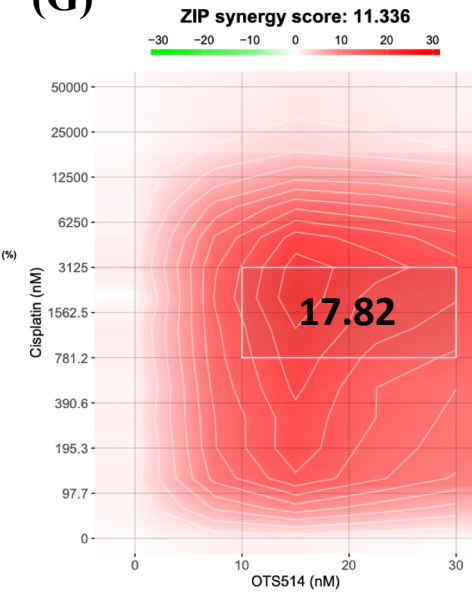**(H)**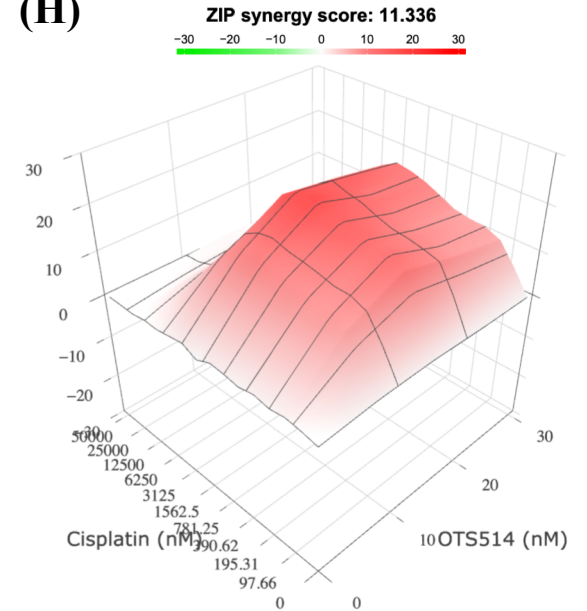

Supplement: Supplementary file 3 — Fig. S3. TOPK inhibitor synergy with cisplatin in osteosarcoma cells. (A) Dose–response curve of cisplatin sensitivity in KHOS treated with different concentrations of OTS514. Cell viability was significantly decreased with increasing OTS514 concentrations. The data show mean ± SD of the independent triple experiment. (B) Dose–response curves and matrix of cisplatin combined with OTS514 in KHOS analyzed by SynergyFinder 2.0. (C) Two‐dimensional synergy map showing additive effect of OTS514 combined with cisplatin in KHOS, with Zero Interaction potency (ZIP) score: 9.945. The most synergistic area in the interaction map was 10 – 30 nM of OTS514 and 390.6 – 1562.5 nM of cisplatin, with ZIP score: 15.35. (D) Three‐dimensional synergy illustration depicting the result from Supplementary Figure 3C. (E) Dose–response curve of cisplatin sensitivity in U2OS treated with different concentrations of OTS514. Cell viability was significantly decreased with increasing concentrations of OTS514. The data show mean ± SD of the independent triple experiment. (F) Dose–response curves and matrix of cisplatin combined with OTS514 in U2OS, analyzed by SynergyFinder 2.0. (G) Two‐dimensional synergy map showing synergistic effect of OTS514 combined with cisplatin in U2OS cells with Zero Interaction potency (ZIP) score: 11.336. The most synergistic area in the interaction map was 10 – 30 nM of OTS514 and 781.2 – 3125 nM of cisplatin, with ZIP score: 17.82. (H) Three‐dimensional synergy illustration depicting the result from Supplementary Figure 3G. *** p<0.001. [file MOL2-15-3721-s004.pdf]
