# Supplementary material for: T‐LAK cell‐originated protein kinase (TOPK): an emerging prognostic biomarker and therapeutic target in osteosarcoma
Source: Mol Oncol. 2021 Jun 29;15(12):3721–37. doi: 10.1002/1878-0261.13039 (PMC8637563; doi:10.1002/1878-0261.13039)
Supplement: Supplementary file 4 — Table S1. Correlations between TOPK expression and clinicopathology in patients with localized osteosarcoma. [file MOL2-15-3721-s001.docx]

**Supplementary Table 1.** Correlations between TOPK expression and clinicopathology in patients with localized osteosarcoma

| **Clinicopathological features** | Number of cases | TOPK expression | | *p* value |
| --- | --- | --- | --- | --- |
|  | (n, %) | Low (n, %) | High (n, %) |  |
| All patients | 53 (100.0) | 10 (18.9) | 43 (81.1) |  |
| Age (years) |  |  |  |  |
| < 20 | 21 (39.6) | 4 (19.0) | 17 (81.0) | 0.943 |
| 20 - 60 | 28 (52.8) | 5 (17.9) | 23 (82.1) |  |
| > 60 | 4 (7.5) | 1 (25.0) | 3 (75.0) |  |
| Gender |  |  |  |  |
| Male | 31 (58.5) | 6 (19.4) | 25 (80.6) | 0.914 |
| Female | 22 (41.5) | 4 (18.2) | 18 (81.8) |  |
| Tumor location |  |  |  |  |
| Femur | 25 (47.2) | 4 (16.0) | 21 (84.0) | 0.337 |
| Tibia | 9 (17.0) | 0 (0.0) | 9 (100.0) |  |
| Humerus | 5 (9.4) | 2 (40.0) | 3 (60.0) |  |
| Pelvis and vertebrae | 7 (13.2) | 2 (28.6) | 5 (71.4) |  |
| Others | 7 (13.2) | 2 (28.6) | 5 (71.4) |  |
| Histologic grade |  |  |  |  |
| Low grade | 9 (17.0) | 2 (22.2) | 7 (77.8) | 0.778 |
| High grade | 44 (83.0) | 8 (18.2) | 36 (81.8) |  |
| Recurrence |  |  |  |  |
| Absent | 35 (66.0) | 9 (25.7) | 26 (74.3) | 0.076 |
| Present | 18 (34.0) | 1 (5.6) | 17 (94.4) |  |
| Metastasis |  |  |  |  |
| Absent | 19 (35.8) | 6 (31.6) | 13 (68.4) | 0.077 |
| Present | 34 (64.2) | 4 (11.8) | 30 (88.2) |  |
| Disease status |  |  |  |  |
| No evidence of disease | 21 (39.6) | 7 (33.3) | 14 (66.7) | 0.029* |
| Alive with disease | 2 (3.8) | 1 (50.0) | 2 (50.0) |  |
| Died of disease | 30 (56.6) | 2 (6.7) | 28 (93.3) |  |
|  |  |  |  |  |

* Statistically significant.
