# Supplementary material for: T‐LAK cell‐originated protein kinase (TOPK): an emerging prognostic biomarker and therapeutic target in osteosarcoma
Source: Mol Oncol. 2021 Jun 29;15(12):3721–37. doi: 10.1002/1878-0261.13039 (PMC8637563; doi:10.1002/1878-0261.13039)
Supplement: Supplementary file 5 — Table S2. Univariate and multivariate OS analysis of prognostic factors in patients with localized osteosarcoma at diagnosis. [file MOL2-15-3721-s005.docx]

| Variable | Overall survival (%) | | | Median overall survival (months) | Univariate analysis | | Multivariate analysis | |
| --- | --- | --- | --- | --- | --- | --- | --- | --- |
|  | 3-year | 5-year | 10-year |  | HR (95% CI) | *p* value | HR (95% CI) | *p* value |
| Age (years) |  |  |  |  |  |  |  |  |
| < 20 | 61.90 | 51.77 | 41.42 | 86.06 | 0.915 (0.49 – 1.71) | 0.780 |  |  |
| 20 – 60 | 67.86 | 67.86 | 60.32 | 170.21 |  |  |  |  |
| > 20 | 66.67 | 33.33 | 33.33 | 54.00 |  |  |  |  |
| Gender |  |  |  |  |  |  |  |  |
| Male | 67.12 | 63.68 | 53.06 | 137.54 | 0.927 (0.45 – 1.91) | 0.837 |  |  |
| Female | 63.64 | 54.21 | 49.28 | 94.25 |  |  |  |  |
| Tumor site |  |  |  |  |  |  |  |  |
| Femur | 60.00 | 56.00 | 48.00 | 93.00 | 0.84 (0.64 – 1.09) | 0.197 |  |  |
| Tibia | 55.55 | 43.21 | 28.81 | 53.40 |  |  |  |  |
| Humerus | 100.00 | 100.00 | 100.00 | 198.00 |  |  |  |  |
| Pelvis and vertebrae | 35.90 | 35.90 | 35.90 | 38.57 |  |  |  |  |
| Others | 100.00 | 85.71 | 68.57 | 132.00 |  |  |  |  |
| Histologic grade |  |  |  |  |  |  |  |  |
| Low grade | 88.89 | 88.89 | 88.89 | 171.75 | 2.49 (0.75 – 8.23) | 0.134 |  |  |
| High grade | 60.90 | 53.68 | 43.92 | 86.04 |  |  |  |  |
| Recurrence |  |  |  |  |  |  |  |  |
| Absent | 71.07 | 68.05 | 58.33 | 264.00 | 2.12 (1.04 – 4.35) | 0.040* | 1.30 (0.62 – 2.70) | 0.487 |
| Present | 55.56 | 44.44 | 38.89 | 60.00 |  |  |  |  |
| Metastasis |  |  |  |  |  |  |  |  |
| Absent | 89.16 | 89.16 | 89.16 | 264.00 | 10.35 (2.46 – 43.55) | 0.001* | 9.61 (2.26 – 40.93) | 0.002* |
| Present | 52.94 | 44.12 | 32.35 | 54.00 |  |  |  |  |
| TOPK |  |  |  |  |  |  |  |  |
| Low expression | 100.00 | 100.00 | 87.50 | 252.00 | 4.48 (1.07 – 18.85) | 0.041* | 4.29 (0.99 – 18.54) | 0.051 |
| High expression | 58.14 | 50.97 | 43.69 | 76.78 |  |  |  |  |

**Supplementary Table 2.** Univariate and multivariate overall survival analysis of prognostic factors in patients with localized osteosarcoma at diagnosis

Abbreviations: *confident interval* (CI), *hazard ratio* (HR), * Statistical significance (*p*<0.05)
